# On the Causes of Baldness

**Published:** 1847-05

**Authors:** 


*On the Causes of Baldness.*—Mr. Cattell, in a paper entitled "Practical remarks on Diseases of the Hair," thus speaks of baldness:

Baldness, in relation to its causes, may be thus distinguished—

First, where it occurs in the vigor of life.

Secondly, where it exists in the decline of life.

In baldness taking place during the vigor of life, we find two opposite conditions of the skin—morbid constriction, and morbid relaxation.

1. Epidermical constriction.

This state of the skin may arise from the powerful influence of the passions, cold, febrile affections, &c.

And it may produce baldness in one of two ways—either by partially strangulating the hairs, and thus preventing the rise of their coloring elements, constituting absolute discoloration; or by causing the hairs to break off on their exit from the skin, leaving behind only the roots of the hairs, but so far hidden and confined below the skin as to constitute baldness.

Such being the one of the immediate causes of baldness, it may, further, be pronounced temporary or permanent.

The duration of baldness resulting from epidermical constriction will be correspondent to the facility or difficulty with which a removal of this state of the skin is accomplished.

Baldness may be only temporary, when the orifices of the hair-pores are closed by dry scales and cells, which frequently collect at their aperture, the hair growing, notwithstanding its inability to escape.

Baldness must be considered permanent when, subsequently to the removal of the epidermical constriction, there exists that condition of the roots of the bulbs, which we shall notice under the head of senile baldness.

The prognosis of baldness is regarded favorable, when the scalp, by friction with the hand, portrays an immediate redness; unfavorable, when the contrary.

2. Epidermical relaxation.

During the continuance of this condition of the skin, the hair not only falls off, but comes out in considerable quantities whenever it is combed or brushed.

Epidermical relaxation, then, becomes a cause of baldness, by permitting an enlargement of the perspiratory pores, and too abundant perspiration; for it is a remarkable fact in persons suffering from the loss of hair through relaxation of the skin, that they perspire on the least exertion or exposure to the heat.

Epidermical relaxation operates, moreover, as a cause of baldness, by destroying the firm interlacement which exists between the roots of the hairs and skin.

That the existence of the hair cannot be permanent, when only held in the skin by its roots, will be evident, if we examine a hair pulled out by its root—in which case, it will be found soft and pulpy in structure. Besides, we must annex the opinion, advanced by many anatomists, that the roots of the hair are fixed in the rete mucosum, or fat, immediately beneath the outer skin.

Associating, then, the existence of epidermical relaxation with the fact that the hairs owe their chief stability to a healthy firmness and condition of the outer skin, it is not in the least surprising that they should be loosened, come out, and leave the patient bald.

Secondly, where it exists in the decline of life.

Baldness in the decline of life does not arise from the two previously mentioned causes, but from a decay of the roots or the bulbs of the hairs.

And it must be observed, that baldness is not an isolated effect of the lapse of years, but a general one, which supervenes in consequence of the obliteration and loss of function that occur in almost all the exterior vessels of the system.

In observing the order of changes giving rise to baldness in aged persons, we may notice,—1st, deficient supply of the colorizing elements of the hair; 2d, death of the internal pulp, the external part only remaining; 3d, after discoloration of the hair has occurred, the small bag which contained the root is gradually obliterated, and finally disappears.—*Lon. Lancet.*—*Ibid.*

